# Symmetrical Catalytic Colloids Display Janus‐Like Active Brownian Particle Motion

**DOI:** 10.1002/advs.202303154

**Published:** 2023-10-23

**Authors:** Richard J. Archer, Stephen J. Ebbens

**Affiliations:** ^1^ Molecular Robotics Laboratory Department of Robotics Graduate School of Engineering Tohoku University Sendai 980‐8579 Japan; ^2^ Department of Chemical and Biological Engineering University of Sheffield Mappin Street Sheffield S1 3JD UK

**Keywords:** active matter, active colloids, catalysis, colloids, Janus particles

## Abstract

Catalytic Janus colloids, with one hemi‐sphere covered by a hydrogen peroxide reduction catalyst such as platinum, represent one of the most experimentally explored examples of self‐motile active colloid systems. This paper comparatively investigates the motile behavior of symmetrical catalytic colloids produced by a solution‐based metal salt reduction process. Despite the significant differences in the distribution of catalytic activity, this study finds that the motion produced by symmetrical colloids is equivalent to that previously reported for Janus colloids. It also shows that introducing a Janus structure to the symmetrical colloids via masking does not significantly modify their motion. These findings could indicate that very subtle variations in surface reactivity can be sufficient to produce Janus‐like active Brownian particle‐type motion, or that a symmetry‐breaking phenomena is present. The study will consequently motivate fresh theoretical attention and also demonstrate a straightforward route to access large quantities of motile active colloids, which are expected to show subtly different phenomenology compared to those with Janus structures.

## Graphical abstract

The motion of symmetrically catalytically active colloids made by platinum salt reduction onto polymer microspheres is observed within hydrogen peroxide solutions. The colloids produce self‐propulsive trajectories that are highly analogous to those observed for catalytic Janus active Brownian particles. The possible mechanistic implications and new applications that this finding could enable are discussed.

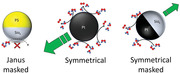

## 1 Introduction

**Figure 1:**
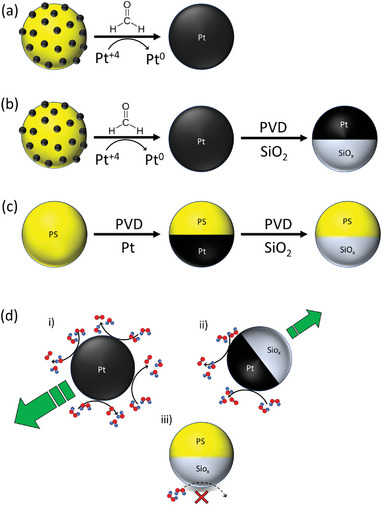
Schematics for the catalytic colloids investigated. (a) Converting Pt seeded colloids to continuous symmetrically Pt coated colloids via Pt salt reduction. (b) Method to convert symmetrically coated Pt coated colloids to masked Janus structure colloids via plasma vapor deposition (PVD) of SiO_2_. (c) Method to investigate the effectiveness of the SiO_2_ mask, by applying a PVD SiO_2_ mask to a PVD deposited Pt Janus coating. (d) Structure of the three colloids investigated here (i) Symmetrical Pt coated colloid (ii) Janus masked symmetrical Pt coated colloid (iii) SiO_2_ masked Pt Janus colloid.

Active colloid research has become a major topic driven by potential applications that can exploit rapid non‐diffusion limited motion,^[^ ^1^, ^2^, ^3^, ^4^ ^]^ augmented interactions^[^ ^5^ ^]^ and associated new collective behavior. Additionally active colloids serve as an ideal theoretical test bed for multi‐component non‐equilibrium interacting systems.^[^ ^6^ ^]^ Significant recent progress has been made, with numerous exciting proof of concept demonstrations including environmental remediation and drug delivery.^[^ ^7^ ^]^ Catalytically active Janus colloids powered by dissolved “fuel” molecules have been a particular focus for experimental research, as they provide relatively easy access to enhanced motion^[^ ^8^ ^]^ and collective phenomena.^[^ ^9^ ^]^ The origin of the experimental realization of catalytic Janus colloids was based on a theoretical description of how asymmetrically catalytically active colloids could generate concentration gradients that would cause them to propel via self‐diffusiophoresis.^[^ ^10^ ^]^ Janus colloids provide an experimentally accessible clearly asymmetrical structure,^[^ ^11^ ^]^ and early experiments indeed confirmed that hemi‐spherically platinum‐coated colloids (e.g., **Figure** **1**c, middle item) decomposing dissolved hydrogen peroxide molecules, undergo enhanced motion.^[^ ^8^ ^]^ The link between the orientation of the catalytic Janus colloid and the direction of propulsion (in this case away from the Pt cap) was also verified, confirming that these colloids are an experimentally accessible example of Active Brownian Particles (ABP).^[^ ^12^ ^]^ Subsequently, a large additional body of experimental and theoretical analysis has taken place for catalytic Janus colloids. While there has been significant mechanistic debate,^[^ ^13^ ^]^ informed by increased experimental understanding: at the heart of each mechanistic proposal for catalytic Janus colloids has been a reliance on the presence of asymmetry to produce motion.^[^ ^14^, ^15^ ^]^

However, mechanisms for self‐sustained motion production via phoresis for symmetrical colloid systems have also been suggested. One analysis showed that self‐sustained motion could result for a symmetrical colloid reliant on the nonlinear interplay between surface osmotic flows and solute advection at specific Péclet numbers.^[^ ^16^ ^]^ An additional example also showed that a uniformly catalytic active sphere could produce motion via reactive fluctuations inducing symmetry breaking in the surrounding fluid.^[^ ^17^ ^]^ The different time‐scales of inertial effects, solute redistribution, and rotational diffusion have also been shown analytically to potentially induce anomalous diffusion phenomena for a symmetrically catalytically active colloid.^[^ ^18^ ^]^ Each of these examples found that specific relative time or length scales are required in order for these new enhanced motion phenomena to manifest. Examination of the citations for these three proposals has not yet revealed any experimental verification for these mechanisms in solid symmetrically active colloids. However elsewhere, literature does reveal signs of a less than critical reliance on a clear Janus structure in order to access enhanced motion.^[^ ^19^ ^]^ These reports mainly concern enzyme‐patterned colloids, which differ from Pt, or other metallic catalytic colloids, due to the discrete surface attachment of each catalytic unit, as opposed to a uniform coating. Indeed, Stochastically Optical Reconstruction Microscopy (STORM) was used in the first report of a non‐Janus urease‐powered micromotor to show that the discrete absorbed catalytic sites were unevenly distributed over the spherical colloids surface.^[^ ^19^ ^]^ It was suggested that the resulting overall asymmetry in catalytic activity drove directed motion. Smaller urease nanomotors, devoid of a Janus structure, have also been used for a range of increasingly sophisticated enhanced diffusion‐based applications.^[^ ^3^, ^20^ ^]^ However, due to their nanometer length scales, it is hard to determine the detailed nature of the trajectories because of rapid Brownian rotation. A recent report also observed directed motion without a Janus structure for photocatalytic micron‐sized spheroidal colloids.^[^ ^21^ ^]^ However, this example also possessed a chemical facet linked reaction asymmetry.

Despite these theoretical and experimental studies findings, the motion of the direct symmetrical analog to Pt catalytic Janus colloids; a micron scale uniformly coated Pt sphere, Figure 1d‐i, in the presence of hydrogen peroxide fuel, has not to our knowledge yet been reported. In contrast to the above experimental studies, a completely reactive Pt‐coated colloid does not have either the discrete catalytic unit absorption feature of enzyme functionalized colloids, or other intrinsic reaction asymmetry, and so more closely resembles the theoretical ideal for a symmetrical catalytic colloid. In this context, here we perform motion analysis for micron‐sized symmetrically Pt‐coated colloids capable of decomposing hydrogen peroxide fuel. Uniform coating is achieved by using a Pt salt‐based solution functionalization to isotropically deposit Pt, Figure 1a. The ability to control the thickness of the deposited Pt layer by varying the salt concentration during coating is verified by sedimentation rate analysis. The colloids are characterized by SEM to confirm the Pt coating integrity and symmetry, and subsequently observed moving freely suspended in hydrogen peroxide containing aqueous solutions. In contrast to our previous study for similar symmetrical Pt‐coated colloids, here we lowered the volume fraction and overall reactivity to avoid the onset of collective convective motion, allowing study under the quiescent conditions required to elucidate the features of individual colloids motion.^[^ ^22^ ^]^ Trajectories for batches of colloids with different thicknesses of deposited Pt are reported, and interpreted via fitting mean‐square displacement against time plots, informed by recent best practice recommendations.^[^ ^23^ ^]^ To ascertain the differences between these uniform catalyst distributions and Janus structures, we also perform comparative trajectory analysis following the addition of a silica mask, Figure 1b.

## 2 Results

**Figure 2:**
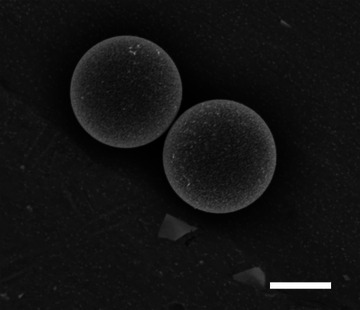
Back scattered Scanning Electron Micrograph for typical Pt coated colloids (40 µL H_2_PtCl_6_), scale bar 2.5 µm.

**Figure 3:**
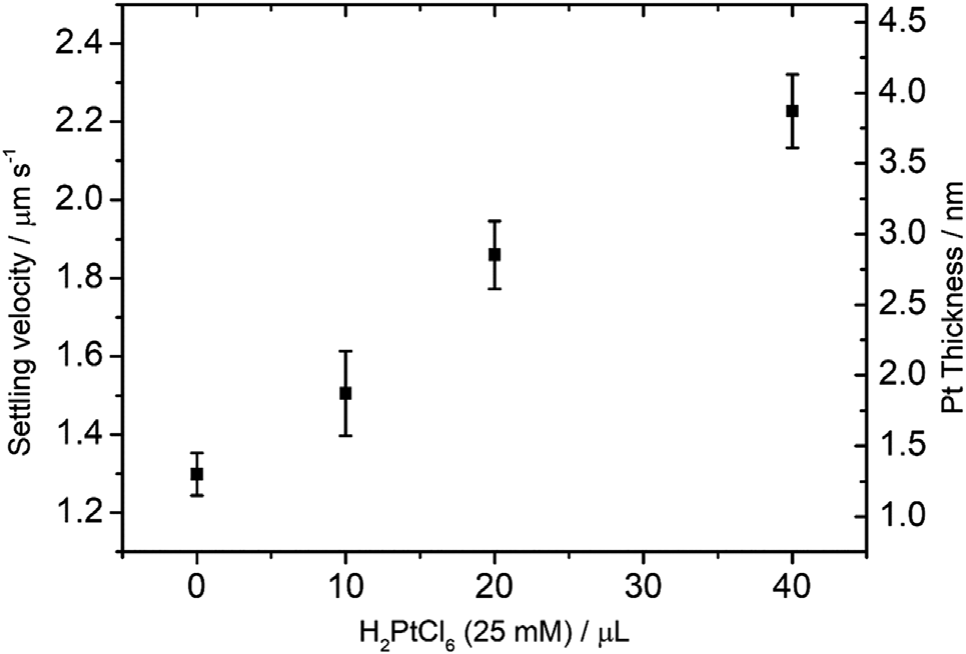
Experimentally observed settling velocities (left) and the corresponding calculated Pt coating thickness values (right).

Batches of symmetrically Pt‐coated PS colloids were prepared by the reduction of varying concentrations of Pt salt‐containing solutions (0‐40 µL H_2_PtCl_6_) in the presence of Kisker supplied Pt seeded PS colloids, as shown schematically in Figure 1a. **Figure** **2** shows a representative back‐scattered scanning electron micrograph (recorded using a FEI Inspect F) for a Pt‐coated colloid batch (40 µL H_2_PtCl_6_). In the imaging mode chosen, contrast is sensitive to variations in composition. The surface of the colloid presents a uniform rough texture, associated with the nanoparticulate nature of the Pt coating, but with no evidence for significant contrast that would indicate regions that have not been coated in Pt. The Pt coating thickness for each batch of functionalized colloids was estimated by measuring the mean sedimentation velocity in water via optical microscopy. According to Stokes’ law, sedimentation velocities are proportional to the density difference between the colloid and the surrounding fluid, and the square of the colloids radius. As a benchmark, the mean sedimentation velocity for uncoated polystyrene colloids measured in this way (0.79 µms^−1^) is consistent with the theoretical prediction for a 4.8 µm diameter particle (0. 77 µms^−1^). Sedimentation velocity increased following the Pt seeding applied to the purchased Kisker colloids, and further increased with Pt salt concentration during the second over‐coating stage, **Figure** **3**. This indicates that higher salt concentrations are causing more metal deposition at the seed particles surface, thereby increasing sedimentation by a combination of the increased overall particle density and radius. By assuming a continuous shell model for the Pt coating/seed layer, it is possible to estimate corresponding Pt coating thicknesses from these sedimentation data. As shown in, Figure 3, this analysis suggests that the purchased nanoparticle decorated colloids have a mean Pt thickness of 1.3 nm, and that this increased to 3.87 nm at the highest salt concentration used (40 µL), with a roughly linear monotonic relationship between available salt concentration and increased Pt thickness. Consequently, the Pt salt reduction method onto Pt‐seeded colloids is demonstrated to be a highly controllable method for making symmetrically coated Pt colloids.

**Figure 4:**
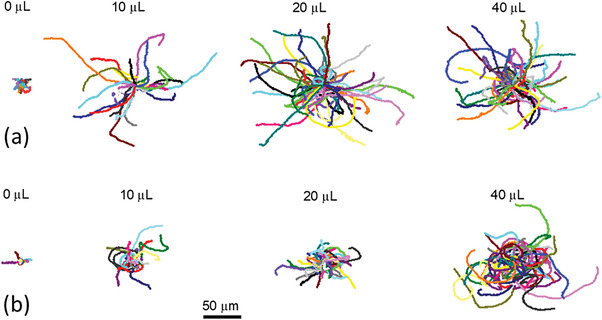
(a) 30 s xy trajectories for symmetrically Pt coated colloids as a function of [H_2_PtCl_6_] during coating stage, displayed on a common scale (b) 30 s xy trajectories for symmetrically Pt coated Janus masked colloids as a function of [H_2_PtCl_6_] during coating stage, displayed on a common scale.

Each batch of symmetrically Pt‐coated colloids was subsequently transferred to a 10% aqueous H_2_O_2_ solution, and observed as a dilute suspension, under an optical microscope. Observations were made in the middle of a small volume cuvette to exclude the influence of confining walls. Video microscopy combined with image analysis was used to generate 2D trajectories for many particles (20<n<64) from each batch, **Figure** **4**a. It is clear that the trajectories are isotropic, with no spatial bias, and also that a significant increase in the average trajectory length occurs for the seeded colloids after further coating via the Pt salt reduction. This change in trajectory was only observed in the presence of 10% hydrogen peroxide: in water the trajectories of all batches are qualitatively indistinguishable, and reflect Brownian diffusion. The progressive increase in trajectory length with increased Pt coating thicknesses suggests that an associated increased catalytic surface activity is modifying the colloids motion, despite the nominally symmetrical Pt coverage.^[^ ^14^ ^]^

**Figure 5:**
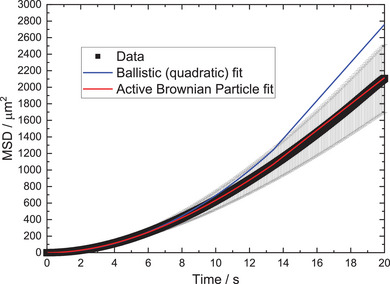
Example MSD v time fit (10 µL H_2_PtCl_6_) comparing the ballistic and Active Brownian Particle models.

In order to understand the colloids’ motion in more detail, we determined the Mean Square Displacements (MSD) as a function of time‐step, Δt averaged over all the colloids observed from each batch. For the size of colloids considered here undergoing slow rotational diffusion relative to the video frame rate, MSD versus time plots are diagnostic of the type of motion. Diffusive or superdiffusive colloids will produce linear MSD v time step (Δt) plots, with a gradient linked to the translational Diffusion coefficient, D_t_ (MSD = 4DΔt). However, ABPs, such as previously reported catalytic Janus colloids,^[^ ^8^ ^]^ show more complex MSD v time behavior, transitioning from short‐term ballistic runs (Quadratic MSD v time) to longer time superdiffusive behavior (Linear MSD v time) due to the coupling between orientation and direction of motion. The timescale for the crossover to diffusive behavior is correspondingly determined by the Brownian rotational diffusion coefficient, D_r_: MSD = 4D_t_Δt+(2v^2^/ D_r_^2^)(D_r_‐1+e^−DrΔt^).^[^ ^8^ ^]^ The MSD versus time plots for each symmetrically coated batch of colloids, are well‐fitted by the equation describing MSD for ABP, **Figure** **5**, and show a clear initial ballistic regime of motion. This suggests that despite their nominal symmetry, these colloids are also well modeled as ABPs, that is, behave as active colloids producing a co‐rotating propulsion velocity vector.

For completeness, we also considered the possibility of buoyancy‐driven convection, which we have previously documented for higher volume fraction concentrations of symmetrically catalytically active colloids.^[^ ^22^ ^]^ However, the signature of convection is qualitatively and quantitatively quite distinct from our observations here. At the length scale of our system, convection does not occur isotopically due to the relationship of flows to the geometry of the container, and so would be apparent in our scatter plots of trajectories as directional motion. Advection would consequently manifest as a purely ballistic MSD versus time plot (MSD = 4D_t_Δt+v^2^Δt^2^),^[^ ^24^ ^]^ which does not fit our results, Figure 5. One further possibility is that these colloids are undergoing bubble propulsion, rather than a phoretic type of propulsion. To discuss this possibility we make a comparison with our previous study, which documented unambiguous bubble propulsive behavior for 30 µm diameter symmetrical platinum‐coated spherical particles.^[^ ^25^ ^]^ Signatures of bubble propulsion observed for these significantly larger colloids include video microscopy resolvable bubble release from the colloidal surface, orders of magnitude faster propulsion velocities (millimeters per second), and chaotic trajectories that could not be modeled by Mean Square Displacement fitting to return plausible values of Brownian diffusion parameters. Moreover, the trajectories for these larger 30 µm colloids showed no link between colloidal orientation and direction of travel, and orders of magnitude shorter persistence lengths than expected for Janus‐type motion. The lack of any of these signatures for bubble propulsion in our observations, leads us to conclude that the much larger curvature in the significantly smaller 5 µm diameter colloids considered in this study is preventing bubble nucleation, as explained theoretically by Fletcher.^[^ ^26^ ^]^ This conclusion is also in agreement with the Zhao group's experimental investigation of the effect of size on ability to nucleate bubbles, which found that 5 µm catalytic platinum colloids were too small to nucleate bubbles, and that colloids needed to be >10 µm in diameter before bubble release and associated motile behavior was observed.^[^ ^27^ ^]^ In summary, this analysis finds two at first surprising results, first that symmetrically functionalized catalytic colloids show clearly enhanced motion in the presence of fuel, and second that this motion appears equivalent to that observed for previous catalytic Janus colloids and fit the ABP model.

In order to further investigate the effect of the distribution of catalytic activity on motion for these colloids, we deposited a hemi‐spherical silica mask using Plasma Vapor Deposition (PVD) onto each batch of symmetrically Pt‐coated colloids, Figure 1b. The aim of the masking layer was to convert the symmetrically coated colloids to a Janus structure in order to ascertain the way this modified the motion. To verify the masks effectiveness, we first applied the Silica mask to a propulsive Pt Janus colloid made via metal evaporation, Figure 1c, and determined the corresponding change in propulsion velocity. This revealed that the mask caused a significant drop in propulsion velocity (from >10 to 2 µms^−1^, see Figure S1, Supporting Information), verifying the ability of the mask layer to substantially reduce the catalytic activity of an underlying Pt layer, Figure 1d‐iii. Figure 4b shows comparative trajectories for each batch of originally symmetrical Pt‐coated colloids following Janus masking. As before, it is clear that depositing additional platinum onto the seeded colloid before masking resulted in an increase in trajectory length. In this case in the Pt salt concentration range 10–20 µL the Janus masked trajectory length over 30 s is less than the equivalent symmetrical colloids; at 40 µL the trajectories appear similar. Qualitatively this suggests that introducing the Janus asymmetry is actually reducing the propulsion velocity for the intermediate thickness Pt coated samples, and having only a small effect on the thickest sample. This suggests, again surprisingly that the distribution of catalytic activity does not have a dramatic affect on colloidal motion.

**Figure 6:**
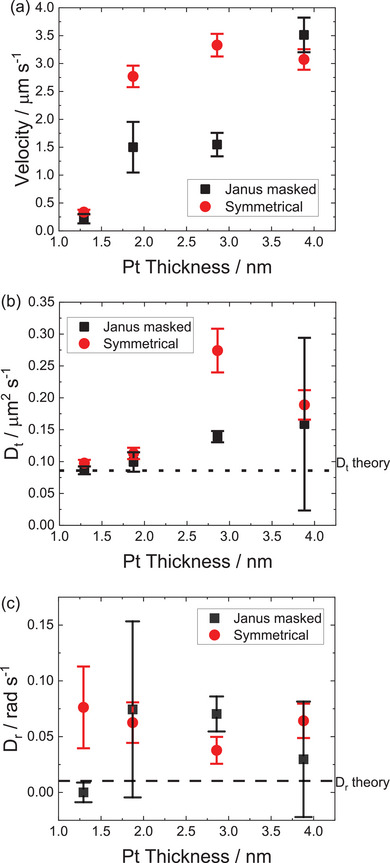
Fitted motion parameters as a function of platinum coating thickness for symmetrically Pt coated, and SiO_2_ masked Janus colloids. (a) Fitted propulsion velocity (b) Fitted translational Diffusion coefficients (c) Fitted rotational Diffusion coefficients. Error bars depict the estimated standard deviation in each parameter, obtained by fitting many bootstrapped averaged MSD v time step curves.

In order to further quantitatively compare each batches’ motion in the masked and un‐masked state, MSD fits to the ABP model were used to determine the key motion parameters. The procedure we used was influenced by the recent analysis of best practice for MSD fitting for active colloids.^[^ ^23^ ^]^ This work suggested that better parameter estimates are obtained by using a weighted least squares fitting method, and avoiding the until now commonplace approach of simplifying fitting by using the “short time limit” version of the ABP MSD equation, which reduces to ballistic behavior when the time fitted is much less than the Brownian rotational time (1/D_r_). In this case, we fitted MSD plots averaged over all available trajectories, to the “full” ABP equation shown above with unconstrained parameters for the first 10 s of MSD data (Fits shown in Figure S2 and S3, Supporting Information). **Figure** **6**a shows the resulting comparison of fitted propulsion velocities. As expected from the trajectories, the symmetrically coated batch shows a significant increase in velocity beyond that for the seeded colloid following the additional coating stage. As the thickness of Pt increases further the velocities remain similar. Whereas for the masked samples, the velocity for the 10 and 20 µL batches are roughly half that of the equivalent symmetrically coated batches. Only at the thickest coated, 40 µL added Pt salt batch, does the Janus masked structure produce a comparable, slightly larger propulsion velocity, than the original symmetrical colloid. These velocity trends confirm that the Janus structure is not conferring significantly higher propulsion velocities, compared to an unmasked structure, and is in fact significantly reducing velocity for intermediate thickness.

For two of the masked samples (10 µL and 40 µL) we noticed a poor MSD v time step fit at longer time periods, due to the presence of oscillations in the MSD, which are a signature of angular propulsion.^[^ ^28^ ^]^ Consequently we fitted these curves to an ABP model that also includes angular propulsion.^[^ ^28^ ^]^ This improved the fit, and suggested values of angular rotation of ω = 0.3 (±0.05) rads^−1^ and 0.22 (±0.01) rads^−1^ for the 10 and 40 µL masked samples, respectively. Similar levels of adventitious angular propulsion have been observed for Pt Janus colloids made by direct metal evaporation, and were assigned to small asymmetries in the hemispherical boundary.^[^ ^8^ ^]^ None of the symmetrical unmasked batches exhibit driven rotations, suggesting that it is the addition of the masking layer that is responsible for the rotations; likely via imperfections at the mask boundary.

Figure 6b shows the fitted D_t_ values for the masked and unmasked batches of Pt‐coated colloids, and the Stokes–Einstein theoretical diffusion coefficient for a 4.8 µm diameter colloid. It is clear that for the Pt seed colloids that have negligible propulsion velocity, D_t_ is in close agreement with theory. As propulsion velocity increases, so does fitted D_t_, possibly due to a correlation in fitting procedure. Note that the significant uncertainty in D_t_ for the 40 µL masked sample reflects the need to fit to a four parameter model to include angular propulsion. Figure 6c shows a similar comparison for fitted D_r_ values. In all batches showing significant propulsion, D_r_ is considerably higher than the theoretical Stokes–Einstein parameter for a 4.8 µm diameter colloid. Other studies of propulsive Janus colloids have also returned D_r_ values that are considerably larger than those predicted by theory.^[^ ^29^ ^]^ There are also significant variations in D_r_, suggesting that extracting accurate D_r_ values from experimental MSD data remains challenging, even adopting recent studies best practice guidelines.^[^ ^23^ ^]^ Again, in particular large uncertainties are seen for the masked samples with angular propulsion (10 µL and 40 µL). In an attempt to refine our experimental method, and reduce errors in the fitted part of the MSD curve, we repeated a subset of our experiments to measure trajectories over several minutes, at the expense of a reduction in spatial resolution due to the larger optical field of view required. However, the fitted motion parameters remained similar to those extracted from 30 s of data. Despite this, the general indication that each propulsive colloid batch is effected by Brownian rotations is nonetheless clear, and fits to models that do not include D_r_ cannot reproduce the features in our MSD data (Figure 5). The addition of the Janus mask does not consistently alter the fitted D_r_ parameters.

## 3 Discussion

Our data has shown that symmetrically coated Pt colloids, expected to have uniform catalytic activity around their surfaces, in fact show very similar Active Brownian Particle like propulsive behavior to previously studied Pt‐Janus colloids. Converting the symmetrical colloids to asymmetrical Janus structures via masking had little effect on the trajectories and Brownian rotation rate, and in fact showed either a dramatic decrease in velocity at intermediate thickness coatings, and only a minor increase for the thickest coated batch. These findings suggest that the introduction of an obvious asymmetrical catalytic distribution in metal catalyst‐based propulsive colloids is not essential. This result should be placed in the context that until now a Janus structure has been viewed as an essential requirement by the large number of catalytic micromotors studies based on Pt and other metallic hydrogen peroxide decomposing metals.^[^ ^11^ ^]^ While there are proposed mechanisms for propulsive motion production by symmetrically catalytic active colloids, highlighted in the introduction, the details of the predicted trajectories and specific parameter requirements would be expected to distinguish these contributions from the ABP type trajectories we report here. As our results show congruence with catalytic Janus colloid like behavior, the origin of ABP motion could potentially be explained via comparison to the explanation of unintended asymmetry used for enzyme functionalized colloids. Additional comparison can be made with the spheroidal photocatalytic BiVO_4_ system introduced above, which is materially homogenous, micron scale, slightly elongated in shape, and produces directed motion powered by decomposing H_2_O_2_.^[^ ^21^ ^]^ While not explicitly modeled as ABPs, the reported trajectories and MSD plots for the BiVO_4_ system appear to possess ABP‐like features. Motion in the Bismuth system is assigned to two factors: spatially segregation of the hydrogen peroxide decomposition by the BiVO_4_ crystalline structure, and the nano and micron sale roughness.

Despite these comparative insights, the colloids described here have less obvious sources of heterogeneity. However, the chemically reduced Pt coating is comprised of fused surface‐bound nanoparticulates, and so is not intrinsically topographically homogeneous at the nm scale, and thickness variations in evaporated Pt coatings have been shown to correlate with surface H_2_O_2_ decomposition rates.^[^ ^14^ ^]^ As an illustration relevant to the local thickness variations that could be present in our current system, changing the thickness of evaporated platinum from 2.5 to 5 nm has been found to increase the reaction rate for H_2_O_2_ decomposition by a factor of 4.^[^ ^14^ ^]^ In light of this discussion, the Janus like ABP motion may arise from small variations in reactivity across each nominally symmetrically Pt‐coated colloid. However, the considerable gap between the structure of the symmetrical colloids that exhibit ABP motion here, and those predicted to produce efficient ABP motion via theory and simulation may warrant additional mechanistic investigation. Due to this discrepancy, it remains a possibility that a symmetry breaking process is instead involved in our observations. However, our system is well below the Péclet number that Michelin et al. proposed would cause spontaneous symmetry breaking (Péclet number, Pe = va/D_solute_, for our system, taking v = 3.25 µms^−1^, a = 2.4 µm and D_H2O2_ = 1.4 × 10^−9^ m^2^s^−1^ Pe = 6 × 10^−3^)^[^ ^16^ ^]^ Our results also suggest that new coating routes may be required to obtain a sufficiently homogenous and spatially symmetrical catalyst distribution to enable the experimental verification of subtle theoretical predictions for idealized uniformly catalytic active colloids motion. However, while there are other methods for patterning catalytic metals onto colloids, these do not appear to give homogeneous coatings, suggesting an unavoidable intrinsic heterogeneity.^[^ ^30^ ^]^ Another possibility is to assess the motion for pure catalytically active objects such as Pt nanoparticles or microspherical Pt particles, which could provide closer analogs to theory. However, these experiments face challenges including the availability of Pt microspheres at suitable sizes, high particle density, and would also change the conductive path, which could in turn have other mechanistic impacts.

Considering the role of Pt thickness on propulsion, it appears that the Pt‐seeded colloids before salt reduction have insufficient Pt‐coating thickness to produce appreciable reactivity, leading to very low velocities. The initial low concentration Pt salt deposition produces a step change in velocity; however, further thickening at higher concentrations does not significantly increase velocity further. This trend indicates that reactivity may rapidly plateau to a maximum value with thickness, as previously reported for evaporatively deposited coatings.^[^ ^14^ ^]^ The reduction in velocity caused by Janus masking for intermediate thickness coated colloids could reflect their reduced overall reactivity. In fact, our control experiment showed the mask was 80% effective, so we would expect a reduction in reactivity via masking to 40%, which is similar to the observed reduction in velocity. For the thickest coated Janus masked sample a slightly higher velocity is produced. This could represent the degree of reactivity at which the efficiency of motion generation via intrinsic coating heterogeneity/symmetry breaking phenomena is surpassed by an imposed Janus structure.

The magnitude of the maximum velocities observed here for symmetrically coated colloids are equivalent to those reported for same‐sized Janus colloids made via metal evaporation of 10 nm thick Pt coatings.^[^ ^31^ ^]^ It is likely that the higher surface area of the nanoparticulate coating used here results in a much lower thickness threshold to produce equivalent reactivity.

Finally, we discuss benefits and future applications for our findings. A key advantage for symmetrically coated colloids is the potential for scalable and straightforward solution phase manufacture. Janus structures are difficult to produce,^[^ ^32^ ^]^ and often achieved only via the use of expensive directional metallization methods. Consequently, the ability to access comparable motion behavior more simply may enable future research and applications. Symmetrically coated ABP like colloids also allow access to subtly different phenomena. For example, gravitaxis seen in Janus colloids will be absent in symmetrically coated systems due to the absence of mass asymmetry.^[^ ^33^ ^]^ Symmetrical active colloids may also conform more closely to angular rotation free ABP motion: here Janus masking introduced noticeable driven rotations, which were not seen for the symmetrical case, and evaporation to a Janus structure also routinely produces batches of active colloids with unavoidable rotations.^[^ ^34^ ^]^ Symmetrical coating consequently allows access to more intrinsically isotropic, and homogeneous active colloid behavior in 2D and 3D.

The lack of a Janus structure will also alter interactions with both the environment and neighboring colloids. Orientation effects, such as alignment in a chemical gradient are not expected due to uniform surface activity and mobility parameters.^[^ ^35^ ^]^ Hydrodynamic fields may also differ from Janus structured colloids, and likewise, chemical field mediated interactions with neighbors will change.^[^ ^5^ ^]^ In these respects, the colloids described here allow may access to some useful new behavior. However, it is likewise clear that motivations to impart Janus structures to active colloids remain, to both enable asymmetry reliant phenomena, and facilitate surface functionalizations, which may be otherwise difficult for completely catalytically coated colloids.

## 4 Conclusion

This study has shown that nominally symmetrically Pt‐coated colloids produce motile behavior powered by catalytic hydrogen peroxide fuel consumption. Trajectory and mean square displacement analysis shows that the resulting motion is highly similar to that observed for Janus structures derived here by masking, and previously reported evaporatively produced catalytic Janus colloids. In all cases, the trajectories are well described as active Brownian particles. This equivalence is surprising given the apparent dramatic difference in surface activity distribution, an observation that may motivate further mechanistic attention. Compared to nominally symmetrical enzyme‐covered colloids and a recent Bismuth colloid study, the heterogeneities driving motion in this Pt‐coated system are more subtle, and suggest that obtaining a truly uniformly symmetrically active colloid may be challenging. Practically, our findings allow straightforward scalable access to active Brownian particles by removing the requirement for a Janus structure. Additionally, symmetrical colloids are expected to differ from catalytic Janus colloids in several respects, including responses to gravity, orientation in chemical fields, and interactions with neighboring particles. All these features have the potential to enable new applications in the active colloid field.

## 5 Experimental Section

### Materials

4.8 µm diameter polystyrene beads coated with platinum as nanoparticles were supplied by Kisker‐Biotechnology. Fluoromax beads 2 µm diameter were supplied by Thermo Fisher Scientific. Hydrogen peroxide (30% wt.%), hexachloroplatinic acid hexahydrate (>37.5% Pt basis), Ethanol (99.8%) and Formaldehyde (37% wt.%) were supplied by Sigma Aaldrich. Deionized (DI) water was obtained from an Elga Purelab Option filtration system (15 MΩ cm). Platinum metal (Pt, 99.99%) and Silica pieces (SiO_2_, 99.99%) used as evaporation sources for physical vapor deposition were bought from Kurt J Lesker.

### Pt Coating

Polystyrene beads (4.8 µm) seeded with a chemically deposited nanoparticulate platinum shell were supplied by Kisker‐Biotech. Further platinum growth was conducted by reducing platinic acid (25 mm, in three different batches with 10 µL, 20 µL and 40 µL of platinic acid added) using formaldehyde (2% wt) in water and ethanol mix solvent (10 ml, 1:1 by volume) in the presence of the Kisker supplied colloids (25 mg ml^−1^, 190 µL), Figure 1a.

### Coating Thickness Analysis

Colloid sedimentation velocities were recorded optically using a horizontal microscope and a Pixelink camera at 33 fps for 30 s with particle tracking performed as described below. Extremely dilute concentrations of colloids were used to prevent colloid–colloid interactions through backflows and to allow each colloid to be treated as an isolated system for the purpose of calculating the velocity due solely to settling. The average thickness of the platinum coatings deposited was quantified as described in the Results section from the overall colloidal density calculated using Stoke's’ law equation for sedimentation velocity (V = 2/9 (ρ″‐ρ)/η gR^2^ where V was the settling velocity, ρ″ was the density of the colloid, ρ was the density of the fluid, η was the viscosity of the fluid, g was acceleration due to gravity, and R was the radius of the settling colloid).

### Particle Tracking

Colloidal motion was digitally recorded at 33 fps using a Pixelink camera (PL‐B742F) attached to a microscope (Nikon eclipse TS‐100). A custom LabView (National Instruments, UK) programme was used to analyze the videos using a pixel intensity‐based threshold algorithm and produced a frame by frame center of mass coordinate as a time referenced x,y output. The analysis of this (x,y,t) trajectory data via calculation of Mean Squared Displacement versus time plots is described in the Results section.

### Janus Masking

Asymmetry was introduced to the symmetrically reactive colloids by coating one hemisphere with an inert mask. Pt‐coated beads were first deposited in a sparse coverage onto glass slides via spin coating (WS650Mz‐23NPPB, Laurell Tech, USA) from ethanol, and then subsequently coated with 30 nm SiO_2_ by electron beam physical vapor deposition under ultra‐high vacuum conditions (Custom PVD chamber, Moorfield, UK), Figure 1b. In the case of the control samples used to verify the masking procedure, PS colloids (Fluoromax, 2 µm diameter, Thermo Fisher Scientific) were spun coat onto glass slides before e‐beam deposition of platinum metal to create catalytically Janus colloids, 30 nm SiO_2_ was subsequently deposited without moving the colloids to directly layer the inert mask on top of the reactive platinum layer, Figure 1c.

## Conflict of Interest

The authors declare no conflict of interest.

## Supporting information

Supporting Information

## Data Availability

The data that support the findings of this study are available from the corresponding author upon reasonable request.
