# Supplementary material for: Symmetrical Catalytic Colloids Display Janus‐Like Active Brownian Particle Motion
Source: Adv Sci (Weinh). 2023 Oct 23;10(33):2303154. doi: 10.1002/advs.202303154 (PMC10667803; doi:10.1002/advs.202303154)
Supplement: Supplementary file 1 — Supporting Information [file ADVS-10-2303154-s001.pdf]

ADVANCED SCIENCE
Open Access

Supporting Information

for *Adv. Sci.*, DOI 10.1002/advs.202303154

Symmetrical Catalytic Colloids Display Janus-Like Active Brownian Particle Motion

Richard J. Archer and Stephen J. Ebbens**

SUPPLEMENTAL INFORMATION:

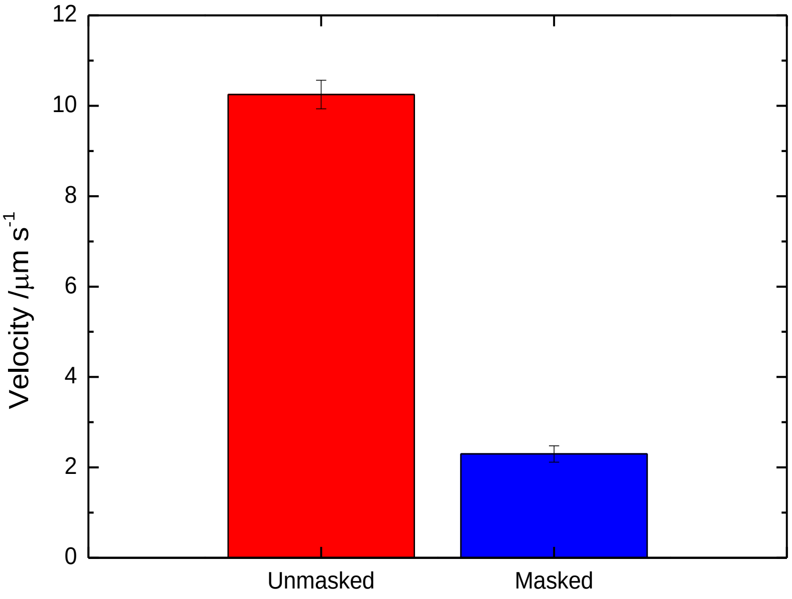

Supplemental Figure 1: Masking effectiveness: comparison of Pt-Janus colloid average fitted velocity before and after masking with SiO_2

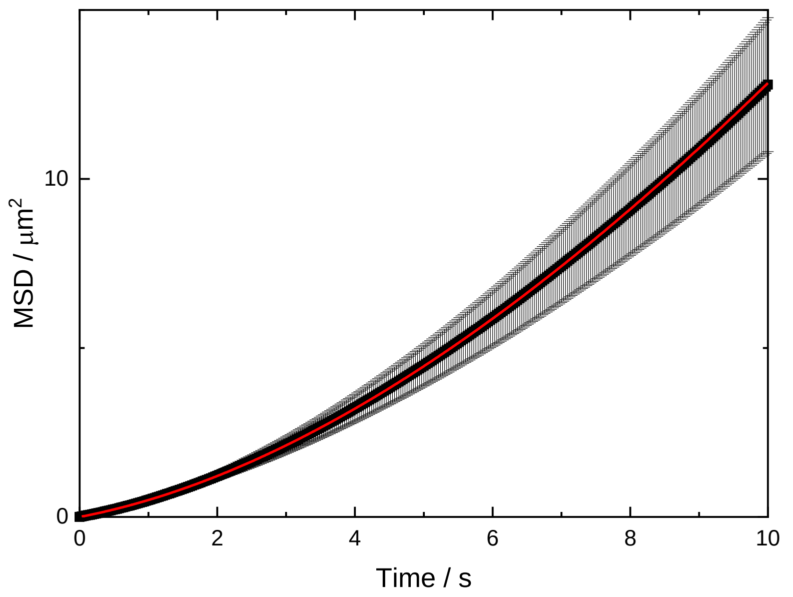

(a) 0 uL Symmetrical Pt MSD fit (n=31)

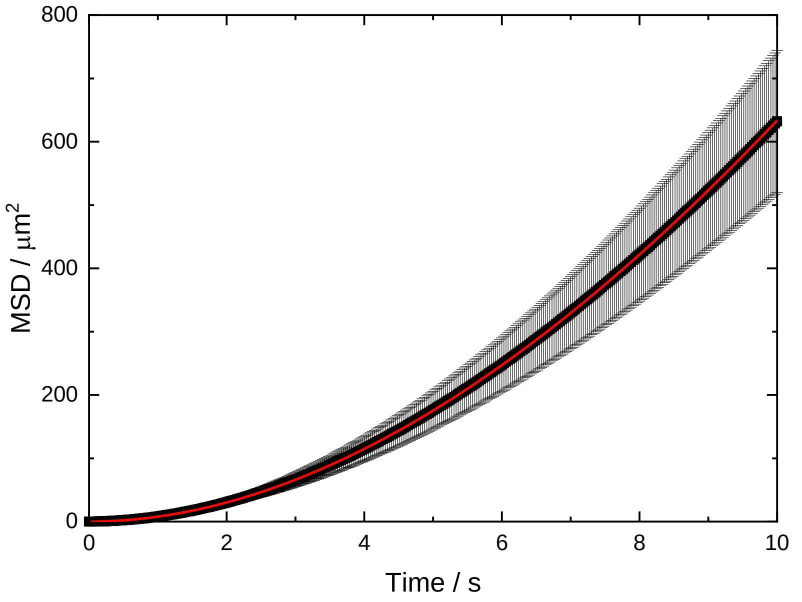

(b) 10 uL Symmetrical Pt MSD fit (n=30)

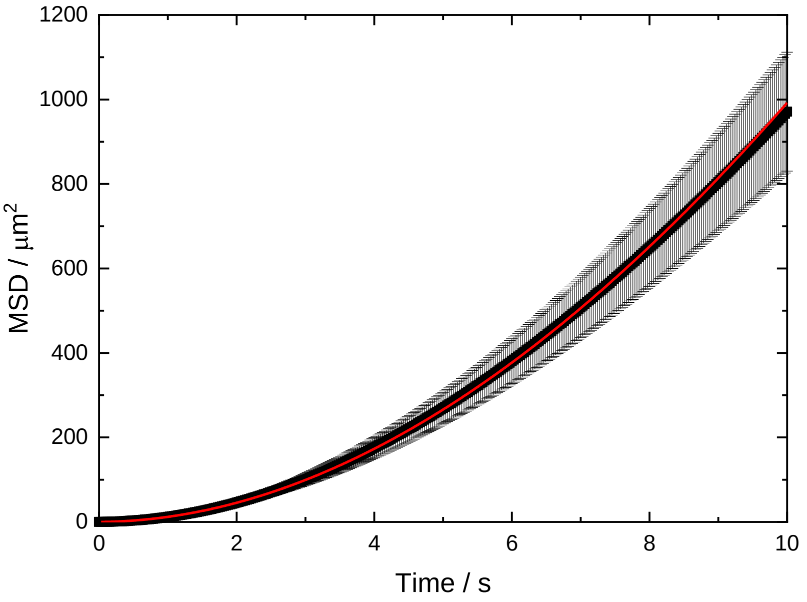

(c) 20 uL Symmetrical Pt MSD fit (n=62)

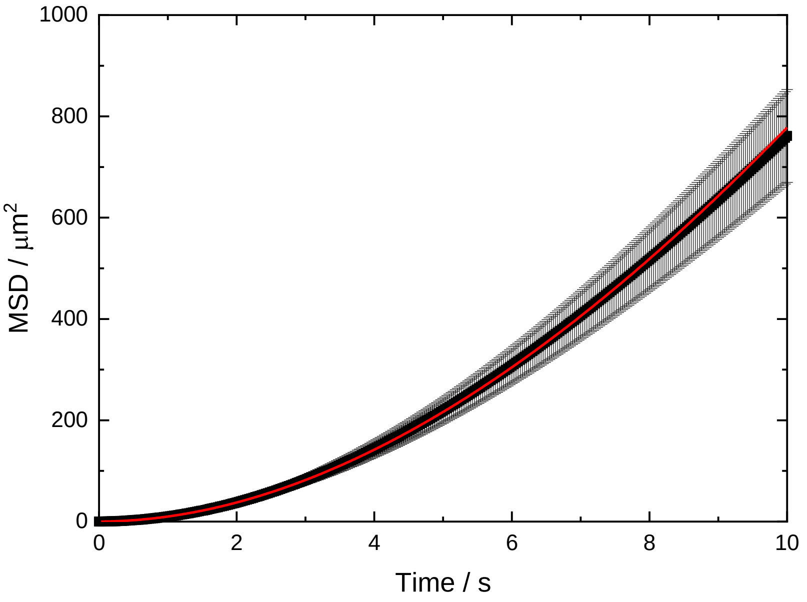

(d) 40 uL Symmetrical Pt MSD fit (n=56)

Supplemental Figure 2: MSD fits for symmetrically coated Pt colloids. Average MSD v time data was obtained by averaging the individual MSD v time data for n colloid trajectories.

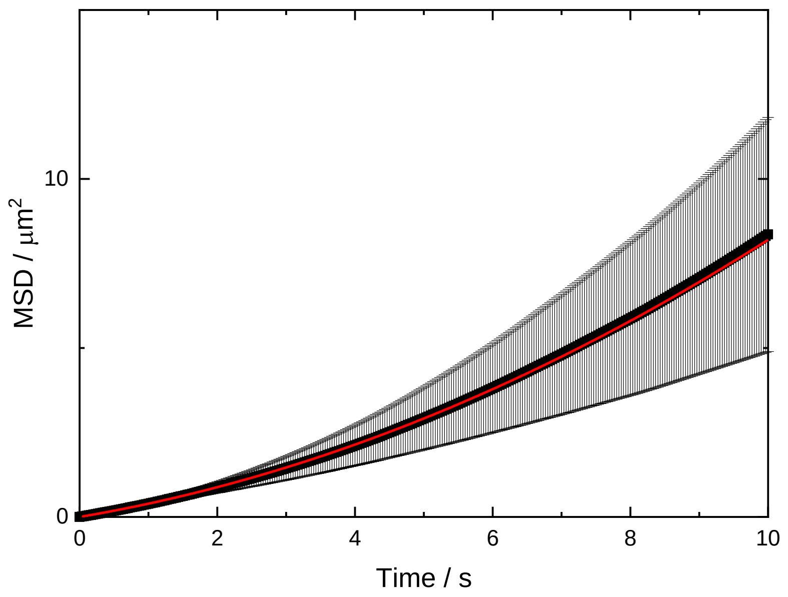

(a) 0 uL Masked Pt MSD fit (n=21)

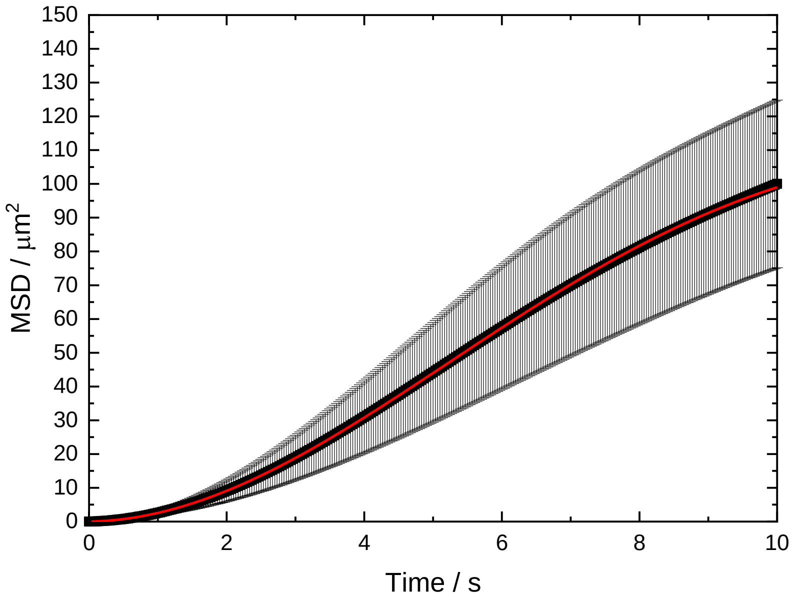

(b) 10 uL Masked Pt MSD fit (n=48) – note this curve is fitted to a model that allows for propulsive angular velocity

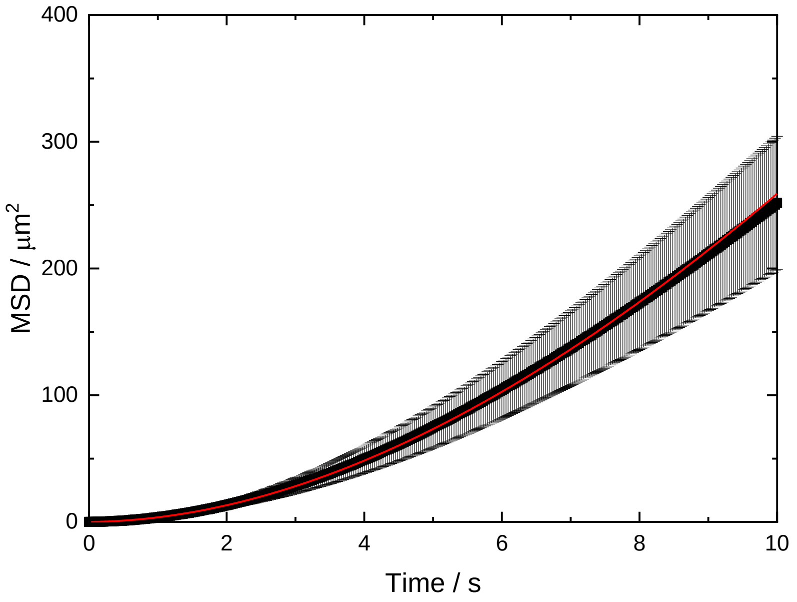

(c) 20 uL Masked Pt MSD fit (n=47)

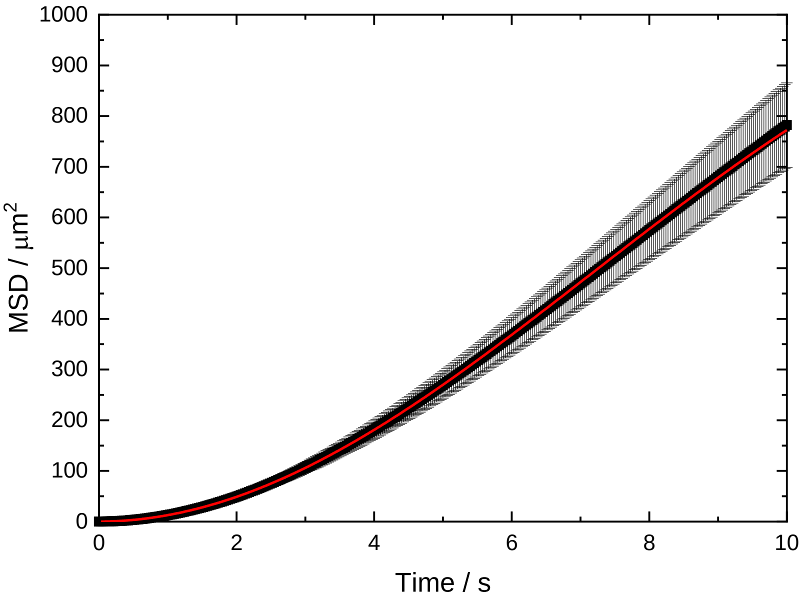

(d) 40 uL Masked Pt MSD fit (n=63) – note this curve is fitted to a model that allows for propulsive angular velocity

Supplemental Figure 3: MSD fits for Janus masked symmetrically coated Pt colloids. Average MSD v time data was obtained by averaging the individual MSD v time data for n colloid trajectories.
